# Multipolar Left Ventricular Lead Implantation in a Unique Coronary Sinus: Direct Drainage of the Posterior Vein into the Right Atrium

**Published:** 2018-04

**Authors:** Ahmet Taha Alper, Mert İlker Hayıroğlu, Hakan Barutça, Ahmet İlker Tekkeşin, Ceyhan Türkkan

**Affiliations:** 1 *Department of Cardiology, Dr. Siyami Ersek Thoracic and Cardiovascular Surgery Training and Research Hospital,* *İstanbul, Turkey.*; 2 *Department of Cardiology, Haydarpasa Sultan Abdulhamid Han Training and Research Hospital, Istanbul, Turkey.*; 3 *Department of Radiology, Dr. Siyami Ersek Thoracic and Cardiovascular Surgery Training and Research Hospital,* *Kadiköy, Istanbul, Turkey.*

**Keywords:** *Heart ventricules*, *Heart atria*, *Coronary sinus*, *Diainage*

## Abstract

The coronary sinus, whose electrical features play an important role in cardiac arrhythmias, is the integral part of the cardiac venous system. Here we describe a 67-year-old male patient with congestive heart failure who was referred to our hospital after the failure of the first cardiac resynchronization therapy defibrillator (CRT-D) implantation. During the cannulation of the coronary sinus, the separate orifice of the posterior cardiac vein was demonstrated by the retrograde filling of the coronary sinus via contrast injection into the posterior cardiac vein. Due to the serious tortuosity of the coronary venous sinus, a multipolar left ventricular lead was implanted using the separate ostium of the posterior cardiac vein. In our patient, the posterior cardiac vein directly drained into the right atrium. At 3 months’ follow-up with the CRT-D, he was asymptomatic (New York Heart Association functional class I).

## Introduction

The coronary sinus, the importance of which has been increasing through its role in providing access for different cardiac procedures, is the integral part of the cardiac venous system. ^1^ The coronary sinus has a complex structure and its electrical features play an invaluable role in invasive arrhythmic procedures. The cannulation of the coronary sinus provides mapping and ablation therapy in many types of arrhythmias. Moreover, over the past decade, the coronary sinus has become a gateway to the left ventricle for biventricular pacing. Augmenting the success rate of left ventricular pacing requires a thorough knowledge of the anatomy of the cardiac venous system. 

## Case Report

**Figure 1:**
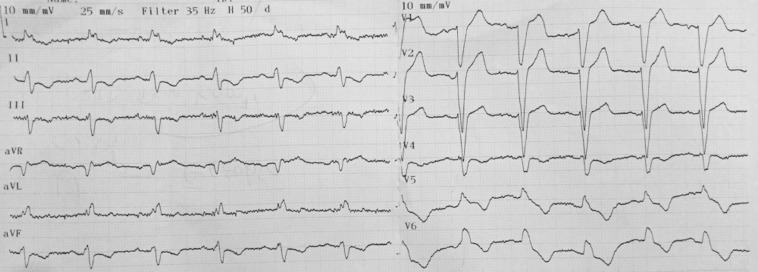
Electrocardiogram before the procedure, presenting sinus rhythm and left bundle branch block with a QRS of 152 ms.

A 67-year-old male patient, who had anterior myocardial infarction and undergone percutaneous coronary intervention on his left anterior descending artery in 2009, was referred to our cardiology clinic after the failure of the first cardiac resynchronization therapy defibrillator (CRT-D) implantation. He had also undergone percutaneous coronary intervention on his right coronary artery 2 years previously. He was diagnosed with a reduced ejection fraction (22%) heart failure after transthoracic echocardiography. His medications included enalapril (10 mg twice daily), carvedilol (25 mg), spironolactone (50 mg), acetylsalicylic acid (100 mg), and ivabradine (7.5 mg). Despite optimal therapy for 8 months, his resting dyspnea and pretibial edema worsened gradually and his exercise capacity was very limited (New York Heart Association functional class III). The 6-minute walking test was performed, and the result was reported to be 180 m. His electrocardiogram (ECG) showed sinus rhythm and left bundle branch block with a QRS of 152 ms (Figure 1)**. **The previous cardiology center attempted to implant a CRT-D in order to palliate the symptoms. Following the failure of the implantation, the patient was referred to our department for reassessment. Another implantation procedure was planned because the patient’s cardiac status sufficiently fulfilled the criteria for CRT-D implantation. 

**Figure 2:**
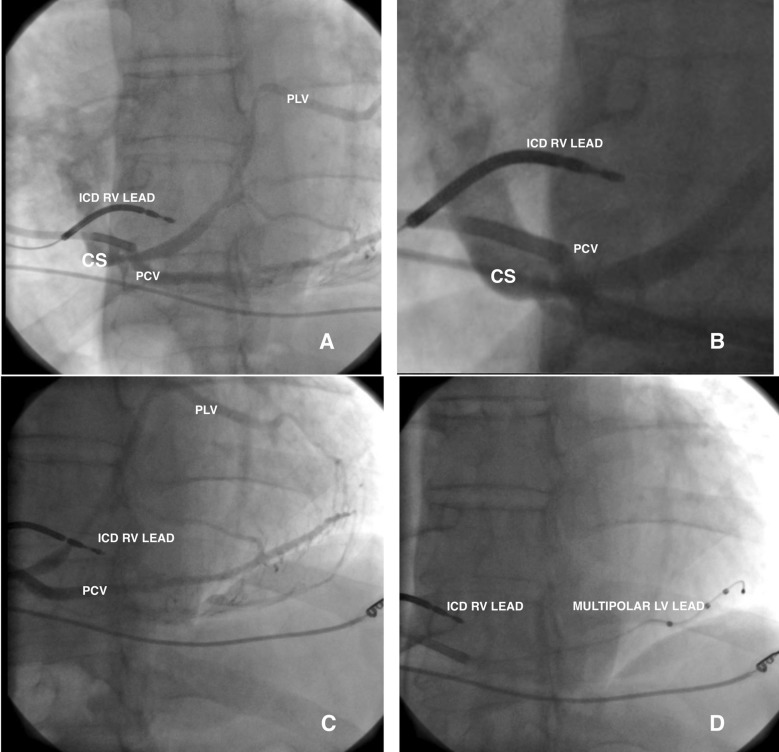
Figure 2A and Figure 2B show the cannulation of the separate orifice of the posterior cardiac vein from the left anterior oblique view of cineangiography.

**Figure 3:**
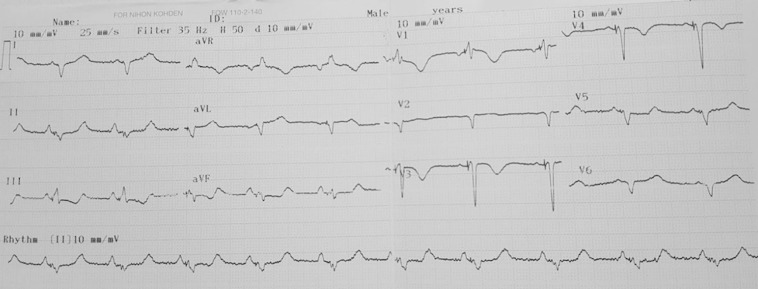
Electrocardiogram after the procedure, presenting sinus rhythm with a narrowed QRS of 152 ms.

**Figure 4:**
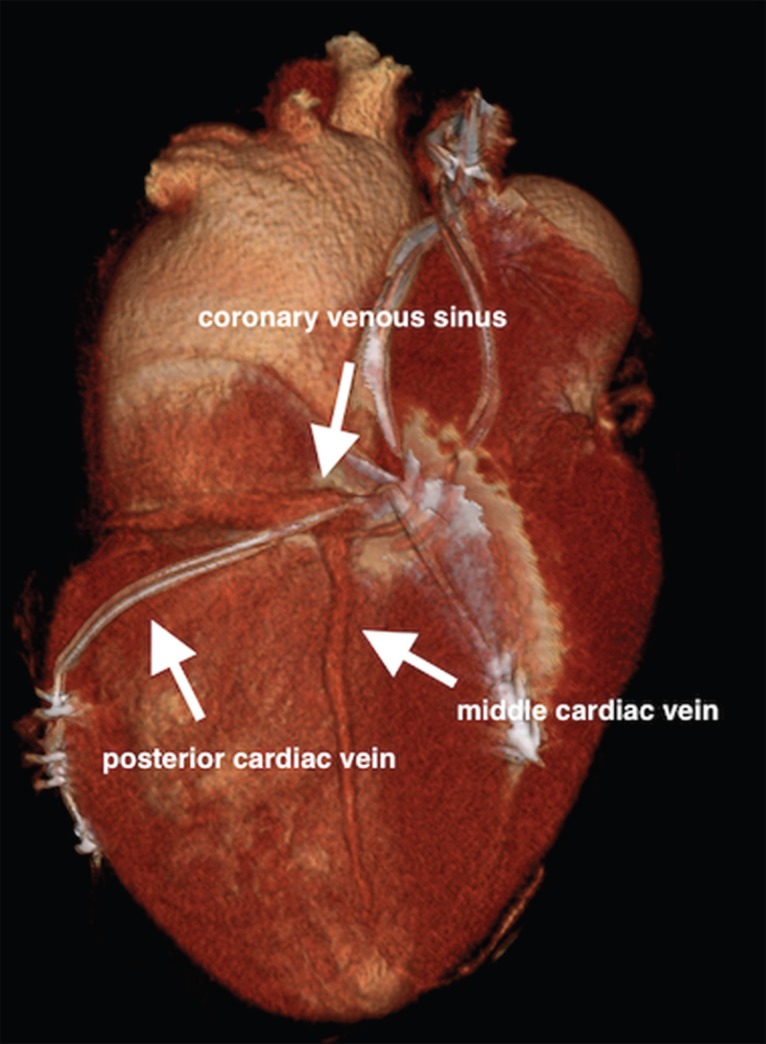
Multi-slice computed tomography reconstruction, showing the coronary venous sinus and the middle cardiac vein. The posterior cardiac vein with a multipolar lead inside is also indicated. The posterior cardiac vein separately drains into the right atrium.

After the cannulation of the coronary sinus with an electrophysiology catheter, a coronary sinus access catheter was placed in the coronary sinus. The angiography of the coronary sinus was performed with a balloon catheter. Efforts were made to place the left ventricular lead in the coronary sinus; however, high tortuosity thwarted the attempts. During maneuvers to detect the middle cardiac vein, the posterior vein was observed to be draining directly into the right atrium (Figures 2A, 2B, and 2C). The posterior cardiac vein was demonstrated to have a separate orifice by the retrograde filling of the coronary sinus via contrast injection into the posterior cardiac vein. A multipolar left ventricular lead was implanted in the posterior vein through the separate ostium from the right atrium (Figure 2D). After the implantation, no complication was detected in the chest X-ray**.** The QRS in the ECG after the procedure narrowed significantly (Figure 3). Verification of the separate ostium was also provided via noninvasive venography using multi-slice computed tomography (Figure 4). 

The patient was stable and he was discharged under the same medical therapy on the fifth postoperative day. At 3 months’ follow-up, he was asymptomatic (New York Heart Association functional class I). The 6-minute walking test was repeated, and the result was reported to be 410 m. Accordingly, to the best of our knowledge, we are the first in the relevant literature to achieve a successful intervention by implanting a multipolar left ventricular lead in a separately originated posterior cardiac vein.

Figure 2B indicates the separate orifice after zooming. Figure 2C illustrates the distal anastomoses of the coronary venous sinus and the posterior cardiac vein from the left anterior oblique view. Figure 2D demonstrates the implantation of the multipolar left ventricular lead by using the separate ostium of the posterior cardiac vein from the left anterior oblique view.

CS, Coronary sinus; ICD, Intracardiac defibrillator; LV, Left ventricle; PCV, Posterior cardiac vein; PLV, Posterolateral vein; RV, Right ventricle

## Discussion

The arterial structure of the heart is well known when compared to venous drainage. The limited knowledge on the venous drainage of the heart renders arrhythmologists unable to solve exceptional problems originating from anatomical variations. The gateway of left ventricular pacing is the coronary sinus, and the coronary sinus system consists of the great cardiac vein with its tributaries. The great cardiac vein is positioned in the atrioventricular sulcus and drains into the posterior portion of the right atrium. The group nearest to the coronary sinus ostium is called “the posterior vein”, the group farthest from the coronary sinus is called “the anterior vein”, and the one located between these two is called “the posterolateral vein”.^2^ There are 5 types of coronary sinus anomalies.^3^ Among these anomalies, separate draining of the posterior cardiac vein ostium has not been described before.

Despite our success in the implantation of the left ventricular lead, our management of the patient was incorrect from one aspect. After failure in the CRT-D implantation, possible reasons should be initially evaluated. One of the most frequent reasons for the failure of the CRT-D implantation is variations in the cardiac venous anatomy. Preoperative noninvasive visualization of the cardiac venous system to determine optimal left ventricular lead positioning was proposed by Veire et al.^4^ Following the failure of left ventricular lead implantation, noninvasive visualization may be performed using multi-slice computed tomography in order to increase success. While cannulating the coronary venous sinus in our patient, we detected anatomical variation. We found the separate orifice of the posterior cardiac vein very suitable for the implantation of left ventricular lead.

## Conclusions

In this report, we described an anatomically unique coronary sinus. We succeeded in implanting a multipolar ventricular lead by using the separate ostium of the posterior cardiac vein and the patient experienced no complication during or after the procedure.

## Notes:

***This paper should be cited as: ***Taha Alper A, Hayıroğlu Mİ, Barutça H, Tekkeşin Aİ, Türkkan C. Multipolar Left Ventricular Lead Implantation in a Unique Coronary Sinus: Direct Drainage of the Posterior Vein into the Right Atrium. J Teh Univ Heart Ctr 2018;13(2):80-83.
